# An Oral Form of Cetylated Fatty Acids versus Meloxicam for Knee Osteoarthritis: A Randomised Clinical Trial

**DOI:** 10.31138/mjr.220823.aof

**Published:** 2023-08-22

**Authors:** Sepide Mohebi, Hamid Reza Farpour, Kayvon Seyed Dehghanian, Sana Sadat Khoshnazar

**Affiliations:** 1 Student Research Committee, Department of Physical Medicine and Rehabilitation, Shiraz University of Medical Sciences, Shiraz, Iran,; 2 Shiraz Geriatric Research Centre, Department of Physical Medicine and Rehabilitation, Shiraz University of Medical Sciences, Shiraz, Iran,; 3 Bone and Joint Diseases Research Centre, Shiraz University of Medical Sciences, Shiraz, Iran

**Keywords:** osteoarthritis, knee, fatty acids, meloxicam, medicine, alternative

## Abstract

**Objective/Aim:**

This study aimed to assess how effective an oral form of Cetylated fatty acids compounds (CFA) is in improving the physical function, pain, and stiffness of individuals suffering from knee osteoarthritis (OA) and how its effectiveness compares to that of Meloxicam, a non-steroidal anti-inflammatory drug (NSAID).

**Methods:**

For this parallel-arm randomised clinical trial, 48 adult patients with knee OA were divided into two groups. The intervention group was prescribed 350mg CFA capsule three times per day for 30 days. The control group was given 15mg of Meloxicam, one tablet daily for ten days. Patients were instructed to fill out the Oxford Knee Score (OKS), Western Ontario and McMaster University Osteoarthritis Index (WOMAC), and Visual Analog Scale (VAS). Data were obtained before the administration of the first dose (considered baseline or t_0_), and two (t_1_), four (t_2_), and eight (t_3_) weeks after the final dose of each intervention.

**Results:**

No significant differences were observed in total WOMAC and OKS scores between the two groups at t_1_, t_2_, or t_3_. However, both groups had significant improvements in their OKS, VAS, and total WOMAC scores compared to their baselines (t_0_). No adverse events were noted in the CFA group.

**Conclusion:**

Improvements in pain intensity and overall physical function were reported in the CFA group. Oral CFAs could safely benefit patients with knee OA.

## INTRODUCTION

Knee Osteoarthritis (OA), a commonly diagnosed degenerative disease of the knee joints, limits mobility and function and impairs quality of life.^1^ Its prevalence is steadily increasing due to the aging of the general population.^2^ The pathophysiology of OA is complex, commonly due to overload or overuse of the joints.^2–4^ Most patients with knee OA seek medical attention, with knee pain and stiffness being their primary complaint.^5^ Exercise and weight loss, supplemented by topical or oral non-steroidal anti-inflammatory drugs (NSAIDs), are the mainstays of OA management.^6, 7^ Surgery is generally reserved only for severe cases.^8, 9^ An astonishing 94% of OA patients have comorbid conditions, with more than half suffering from cardiovascular diseases.^10^ Meloxicam, an NSAID and a selective inhibitor of cyclooxygenase-2 (COX-2), especially at lower doses, has a better side effect profile than other NSAIDs;^11^ however, adverse cardiovascular and renal events are still likely.^12, 13^

In recent decades, new and potentially safer treatments such as mesotherapy, prolotherapy, viscosupplementation, and cetylated fatty acids compounds (CFA) have been proposed for managing knee OA.^14–16^ The efficacy of the topical forms of CFA in treating OA has been assessed in several studies.^17, 18^ Studies on oral forms of CFA in the treatment of OA have been lacking.

This paper focuses on an oral form of CFA, a blend of esterified fatty acids that help decrease inflammation-induced pain.^19^ This study aimed to assess how effective an oral form of CFA is in improving the physical function, pain, and joint stiffness of individuals suffering from knee OA and how its effectiveness compares to that of Meloxicam.

## MATERIALS AND METHODS

### Trial Design

**Figure 1.**
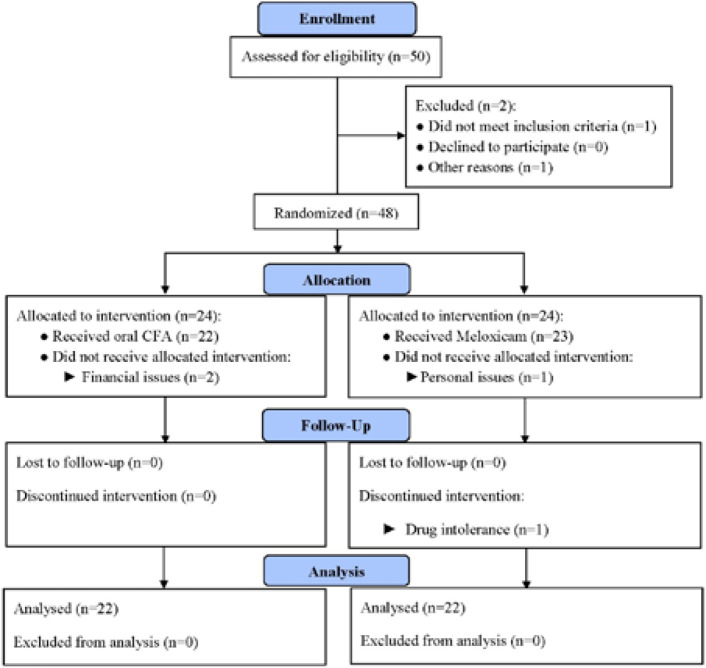
CONSORT flowchart of the trial.

This parallel-arm randomised clinical trial was single-blinded. Only investigators and statisticians were blinded. Due to differences in the physical appearance and duration of use of each treatment, it was not possible to blind participants. **Figure 1** illustrates the enrolment, allocation, follow-up, and analysis flowchart.

### Sample Size

With an expected dropout rate of 10% and considering a power of 0.8, and a significance level of 0.8, the necessary sample size was 24 patients in each group.

### Selection Criteria

The inclusion criteria were as follows: being aged 35–70 years old; meeting the clinical criteria for knee OA according to the American College of Rheumatology;^20^ being classified as grade 2 (minimal) or 3 (moderate) according to the Kellgren and Lawrence classification system;^21^ having symptoms of knee pain, stiffness, and crepitus for at least three months.

Patients with preexisting conditions with possible overlapping pain symptoms were excluded from the study. These conditions included, cancer, diabetes mellitus, rheumatologic diseases of the knees, trauma to the knees during the previous three months, prior total knee replacement surgery.^15^ Pregnant patients and patients with bleeding disorders, acute radiculopathy, and gastro-intestinal intolerance were also excluded from the study due to safety concerns.

### Randomisation and Blinding

The clinic’s administrator randomly allocated forty-eight eligible subjects to two parallel groups using a block randomisation list. A computer compiled a non-stratified list with a block size of four. Statisticians were blinded to the allocation.

### Intervention

Oral CFA and Meloxicam were our two medications. Medication costs were covered by the patients. The intervention group was prescribed Rheumatidin™, 350mg CFA capsule from Webber Naturals (British Columbia, Canada), three times per day for 30 days according to its treatment protocol. The control group was given Meloxicam, 15mg from Irandaroo (Barkat Pharmaceutical Group, Tehran, Iran), one tablet per day for ten days. The shortest duration of treatment that achieved the anti-inflammatory and analgesic effects of NSAIDs was selected.^22^ We prescribed Acetaminophen tablet (325mg) as a rescue drug for both groups to be used in case of severe pain. An advantage of allowing a rescue medication is determining the efficacy of the prescribed intervention in OA-related pain management. Patients were advised to stop all other therapies for knee OA.

### Outcomes

Patients were instructed to fill out the Oxford Knee Score (OKS),^23^ Western Ontario and McMaster University Osteoarthritis Index (WOMAC),^24^ and Visual Analog Scale (VAS).^25^ Demographic information such as age and gender were collected. The Oxford Knee Score (OKS) is a 12-item questionnaire in which patients answer questions on a 0–4 ordinal scale. The sum of the scores gives a total OKS score that ranges from 0–48. Higher scores represent better conditions. The WOMAC questionnaire inquires about Pain (five items), Stiffness (two items), and Physical Function (17 items). Each item is scored on a 5-point scale ranging from zero (None) to four (Extreme). The scores for each domain are then added up. The total WOMAC score ranges from 0–96 and is calculated by summing up the scores of all three subscales. Higher scores represent worse conditions. The Persian translations of OKS and WOMAC have demonstrated high validity and good reliability for assessing osteoarthritic knee pain.^26,27^ Pain intensity was measured on the Visual Analog Scale (VAS). Zero on the VAS means no pain; ten indicates the worst possible pain. Data were obtained before the administration of the first dose (considered baseline or t_0_), and two (t_1_), four (t_2_), and eight (t_3_) weeks after the final dose of each intervention. A second colleague, blinded to the patients’ assigned groups, helped fill out the questionnaires and noted any side effects.

### Statistical Method

Data are presented as mean ± standard deviation (SD). SPSS software version 25.0 (Armonk, New York: IBM Corp) was used for all analyses. The statistical tests used were the repeated measure ANOVA, independent-samples t-test, paired-samples t-test, Chi-square, and Fisher’s exact test. P-values less than 0.05 were considered statistically significant.

### Safety

Tolerability and adverse events were noted during each visit. The research team then followed up and fully treated any complications or side effects.

## RESULTS

A total of 50 patients were assessed, of whom 48 met the eligibility criteria. Twenty-four patients were randomly allocated to each group. Four patients were excluded during the study (**Figure 1**), and the results of the final 44 patients are presented here.

The CFA group consisted of 6 male (27.3%) and 16 female (72.7%) participants with a mean age of 50.4 ± 9.2. Seven male (31.8%) and 15 female (68.2%) patients were allocated to the Meloxicam group with a mean age of 47.7 ±7.7. The two groups did not differ significantly based on age (P-value=0.30) or gender (P-value=0.74).

**Figure 2.**
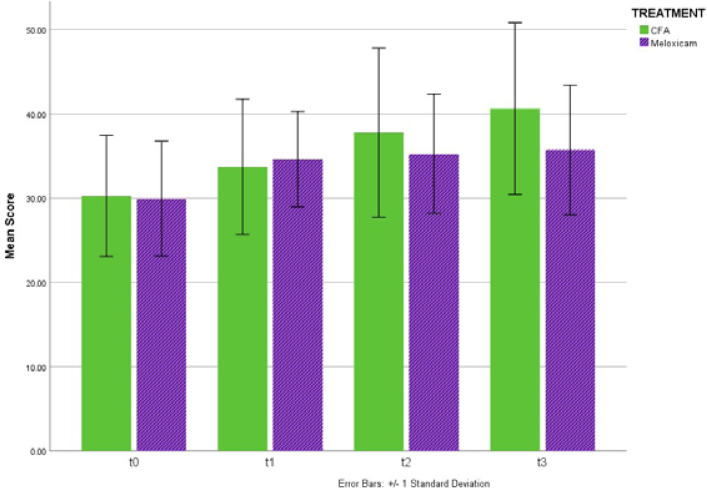
Oxford Knee Score (OKS) scores in people using oral cetylated fatty acids (CFA) or Meloxicam at t0, t_1_, t_2_, and t_3_.

Differences in mean OKS scores between the two groups failed to reach statistical significance at t_1_, t_2_, and t_3_ (**Table 1**); However, in both groups, improvements were observed in OKS scores compared to their baselines (t_0_) (**Figure 2**).

**Table 1.** Comparisons of Visual Analog Scale and Oxford Knee Score between groups.

**Variables**	**Time**	**CFA**		**Meloxicam**		**P-value between groups**
		**Value (mean ± SD)**	**Change from Baseline (mean ± SD)**	**Value (mean ± SD)**	**Change from Baseline (mean ± SD)**	
**VAS^a^**	t_0_	7.14 ± 1.70	N/A^b^	7.82 ± 1.01	N/A^b^	P=0.11^*^
	t_1_	5.36 ± 2.06	−1.77 ± 1.15, P=0.0000004^**^	6.05 ± 1.50	−1.77 ± 1.15, P=0.0000004^**^	P=0.21^*^
	t_2_	3.91 ± 2.74	−3.23 ± 1.82, P=0.00000004^**^	5.50 ± 1.44	−2.32 ± 1.40, P=0.0000001^**^	P=0.02^*^
	t_3_	2.59 ± 2.28	−4.55 ± 1.82, P=0.0000000001^**^	5.14 ± 1.70	−2.68 ± 1.96, P=0.000002^**^	P=0.0001^*^
**OKS^c^**	t_0_	30.27 ± 7.19	N/A^b^	29.95 ± 6.82	N/A^b^	P=0.88^*^
	t_1_	33.73 ± 8.04	3.45 ± 5.38, P=0.007^**^	34.64 ± 5.66	4.68 ± 3.59, P=0.000005^**^	P=0.67^*^
	t_2_	37.77 ± 10.03	7.50 ± 7.91, P=0.0002^**^	35.27 ± 7.07	5.32 ± 4.38, P=0.00001^**^	P=0.35^*^
	t_3_	40.64 ± 10.18	10.36 ± 8.21, P=0.000007^**^	35.73 ± 7.70	5.77 ± 4.12, P=0.000002^**^	P=0.07^*^

a Visual Analog Scale;

b Not Applicable;

c Oxford Knee Score;

* Independent-samples T-test;

** Paired-samples T-test

**Figure 3.**
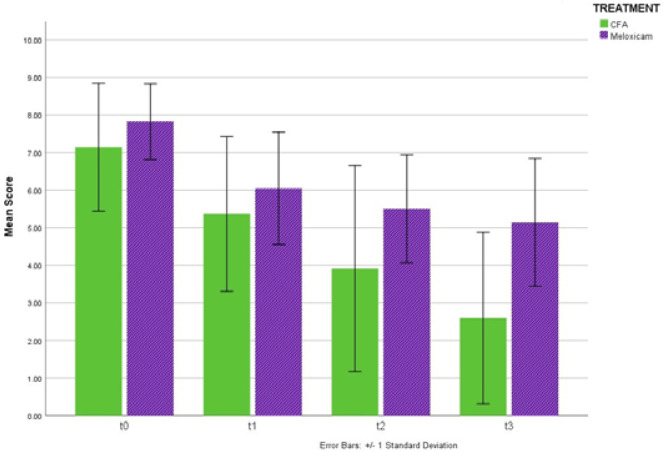
Pain intensity as measured on the Visual Analog Scale (VAS) in patients using oral cetylated fatty acids (CFA) or Meloxicam at t0, t_1_, t_2_, and t_3_.

**Figure 4.**
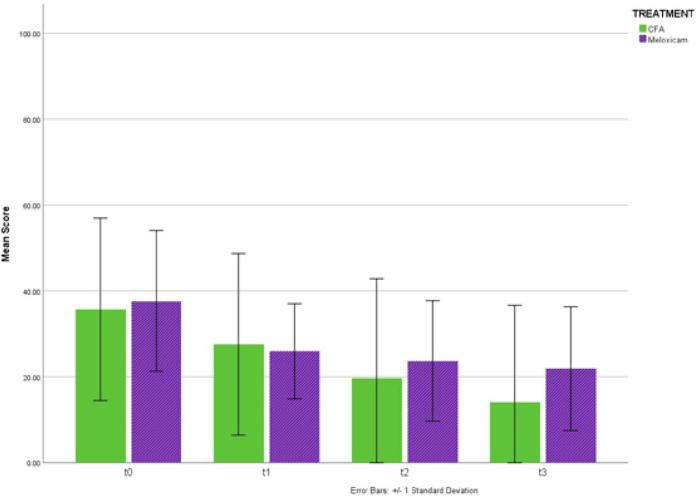
Western Ontario and McMaster University Osteoarthritis Index (WOMAC) scores in participants using oral cetylated fatty acids (CFA) or Meloxicam at t0, t_1_, t_2_, and t_3_.

Both groups experienced pain reductions as measured on the VAS compared to their baselines (t_0_) (**Figure 3**). Compared to t_0_, VAS scores had decreased by 24.9% (P=0.0000004) by week two, 45.2% (P=0.00000004) by week four, and 63.7% (P=0.0000000001) by week eight in the CFA group. Patients in the CFA group had significantly less VAS scores at t_2_ (P=0.02) and t3 (P=0.0001) than those in the Meloxicam group (**Table 1**). Improvements in total WOMAC scores were observed at two, four, and eight weeks following the final dose in both groups compared to t_0_ (**Figure 4**). There were no statistically significant differences in mean total WOMAC scores between the two treatment groups at t_1_, t_2_, and t_3_. **Table 2** shows comparisons of WOMAC scores between the two groups.

**Table 2.** Comparisons of Western Ontario and McMaster University Osteoarthritis Index and its subscales scores between groups.

**Variables**	**Time**	**CFA**		**Meloxicam**		**P-value between groups**
		**Value (mean ± SD)**	**Change from Baseline (mean ± SD)**	**Value (mean ± SD)**	**Change from Baseline (mean ± SD)**	
**Pain**	t_0_	8.18 ± 4.91	N/A^a^	9.82 ± 3.58	N/A^a^	P=0.21^*^
	t_1_	5.91 ± 4.26	−2.27 ± 2.88, P=0.001^**^	6.50 ± 2.32	−3.32 ± 2.40, P=0.000002^**^	P=0.57^*^
	t_2_	3.91 ± 4.48	−4.27 ± 4.27, P=0.0001^**^	5.82 ± 3.25	−4.00 ± 3.16, P=0.000007^**^	P=0.11^*^
	t_3_	2.64 ± 4.08	−5.55 ± 4.80, P=0.00002^**^	5.41 ± 2.86	−4.41 ± 2.52, P=0.00000005^**^	P=0.01^*^
**Stiffness**	t_0_	1.32 ± 1.78	N/A^a^	1.10 ± 1.38	N/A^a^	P=0.64^*^
	t_1_	0.91 ± 1.23	−0.41 ± 0.85, P=0.03^**^	0.82 ± 1.26	−0.27 ± 0.70, P=0.08^**^	P=0.81^*^
	t_2_	0.95 ± 1.96	−0.36 ± 1.60, P=0.29^**^	0.55 ± 0.96	−0.55 ± 1.01, P=0.01^**^	P=0.39^*^
	t_3_	0.55 ± 1.26	−0.77 ± 1.38, P=0.01^**^	0.50 ± 0.91	−0.59 ± 1.01, P=0.01^**^	P=0.89^*^
**Physical Function**	t_0_	26.27 ± 15.45	N/A^a^	26.73 ± 12.48	N/A^a^	P=0.92^*^
	t_1_	20.77 ± 16.26	−5.50 ± 8.08, P=0.004^**^	18.68 ± 8.75	−8.05 ± 6.81, P=0.00001^**^	P=0.59^*^
	t_2_	14.86 ± 17.92	−11.41 ± 12.11, P=0.0002^**^	17.09 ± 10.99	−9.64 ± 6.86, P=0.000002^**^	P=0.62^*^
	t_3_	11.00 ± 17.53	−15.27 ± 13.56, P=0.00003^**^	16.05 ± 11.68	−10.68 ± 6.45, P=0.0000001^**^	P=0.27^*^
**WOMAC^b^** **Total**	t_0_	35.77 ± 21.21	N/A^a^	37.64 ± 16.38	N/A^a^	P=0.75^*^
	t_1_	27.59 ± 21.13	−8.18 ± 10.89, P=0.002^**^	26.00 ± 11.07	−11.64 ± 9.33, P=0.000008^**^	P=0.76^*^
	t_2_	19.68 ± 23.12	−16.09 ± 16.17, P=0.0001^**^	23.73 ± 14.02	−13.91 ± 9.87, P=0.000002^**^	P=0.49^*^
	t_3_	14.18 ± 22.52	−21.59 ± 18.76, P=0.00002^**^	21.95 ± 14.42	−15.68 ± 8.56, P=0.00000002^**^	P=0.18^*^

a Not Applicable;

b Western Ontario and McMaster University Osteoarthritis Index;

* Independent-samples T-test;

** Paired-samples T-test

Six patients (27.2%) in the CFA group used Acetaminophen for severe pain during the first two weeks. None (0%) used the rescue drug after week 2. No Acetaminophen consumption was reported in the Meloxicam group. No adverse events were observed or reported during the trial in those treated with oral CFA. One patient in the Meloxicam group reported poor drug tolerance due to dyspepsia, so they were excluded from the trial.

## DISCUSSION

Our study aimed to assess whether oral CFA effectively reduces joint pain, stiffness, and improves physical function and whether it could be used as an alternative to Meloxicam for patients suffering from knee OA. We found no significant differences in total WOMAC and OKS scores between the two treatment groups at t_1_, t_2_, or t_3_. However, both groups had significant improvements in their OKS, VAS, and total WOMAC scores compared to their baselines (t_0_).

Knee OA, the most common form of OA, is a chronic degenerative disease with symptoms of pain, stiffness, and decreased joint motion. At the time of diagnosis, patients live an average of 30 years with this disease.^28^ There has been a major shift toward non-pharmacologic treatment of knee OA, but topical or oral NSAIDs are still considered the pharmacological therapy of choice.^29^ Researchers have sought alternative pharmacologic treatments for the management of OA. The use of CFA has been proposed. CFAs reduce inflammation in vitro by decreasing the production of inflammatory regulators such as TNF, IL-6, and MCP-1.^19^ When used orally or as a topical cream, CFAs have been shown to improve balance, stair-climbing ability, chair “up-and-go” performance, knee range of motion,^18^ and static postural stability.^30^

In the group treated by oral CFA, we observed improvements in physical function, as measured by the subscale of the WOMAC questionnaire, at t_1_, t_2_, and t_3_ compared to t_0_. Other studies have reported similar findings. Hesslink et al.^31^ found that consuming 350mg of oral CFA for 68 days improves knee flexion and overall function in patients with knee OA. Kraemer et al.^32^ showed that a blend of CFA and menthol used topically twice daily for seven days reduces pain and improves functional performance in individuals with knee OA. Therefore, we postulate that CFA can help overcome the functional limitations brought on by knee OA.

Patients in the oral CFA group reported less pain on the VAS and the “Pain” subscale of the WOMAC questionnaire, at t_1_, t_2_, and t_3_ compared to t_0_. Similarly, in a study by Udani et al.,^33^ CFA, as compared to placebo, significantly lowered pain as measured on the VAS after eight weeks of treatment. These findings support the claim that oral CFA could be a promising treatment for pain reduction in knee OA patients. However, we believe that, as revealed by Kraemer et al.,^18^ treatment with CFA has a rather chronic and cumulative effect. This chronic treatment effect of oral CFA might explain why almost a quarter of patients in the CFA group had used the rescue drug due to severe pain during the first two weeks of the trial.

Our study found no clinically significant adverse events in the CFA group. Research suggests that, even at high doses, oral CFAs are safe and non-toxic for the general adult population.^33–35^ Hence, we believe oral CFAs could be safe, novel treatments for knee OA.

### Study Limitations

Our study had some limitations:

1. We found that the use of oral CFA, as opposed to Meloxicam, resulted in a more significant reduction in pain intensity at t_3,_ as reported on the VAS and the “Pain” subscale of the WOMAC questionnaire. However, this finding should be interpreted with caution. According to the study protocol, the duration of treatment with Meloxicam was set to ten days, but studies show that at least three weeks of use is needed to reach the full anti-inflammatory effect of NSAIDs.^22^ Moreover, NSAIDs have a short carry-over effect, and following treatment cessation, osteoarthritic pain increases rapidly.^36^
2. Results may be biased since patients in this trial were not blinded.
3. Although a trained colleague helped patients fill out the questionnaires, patients’ responses were self-reported and might not reflect reality.
4. We only assessed the short-term effects of oral CFA on knee OA. Future studies can evaluate this treatment’s long-term effectiveness in managing knee OA.

## CONCLUSION

Although no significant differences were observed between the OKS and total WOMAC scores of the two treatment groups, improvements in pain intensity and overall physical function were reported in the CFA group. We postulate that oral CFAs could safely and effectively benefit patients with knee OA.

## TRIAL REGISTRATION

This study was registered at the Iranian Registry of Clinical Trials as a clinical trial with the registration code: IRCT20180629040282N1 on 4/18/2019.

## PARTICIPANTS

Patients referred to the physical medicine and rehabilitation clinics of Emam Reza Clinic in 2019 were recruited for the study. The clinics are academic centres affiliated with Shiraz University of Medical Sciences, Shiraz, Iran.

### Ethical Considerations

This study was registered at the Iranian Registry of Clinical Trials (available at https://irct.ir/trial/33476) as a clinical trial with the registration code: IRCT20180629040282N1. The Medical Ethics Committee of Shiraz University of Medical Sciences (SUMS) approved the study under reference number “ir.sums.med.rec.1397.238”. The researcher obtained written informed consent and explained the study’s objective to each participant before enrolment in the trial. Participation was voluntary, and the patients were allowed to withdraw from the trial at any stage. The methods and design of the study were unchanged after the commencement of the trial. Acetaminophen was prescribed as a rescue drug to be used in case of severe pain.

## Data Availability

The authors declare that the data supporting the findings of this study are available within the article. All other data are available from the corresponding author upon reasonable request.

## TRIAL REGISTRATION

This study was registered at the Iranian Registry of Clinical Trials (available at https://irct.ir/trial/33476) as a clinical trial with the registration code: IRCT20180629040282N1 on 4/18/2019.

## ABBREVIATIONS

CFA: Cetylated fatty acids
NSAIDs: Non-steroidal anti-inflammatory drugs
OA: Osteoarthritis
OKS: Oxford Knee Score
SD: Standard deviation
VAS: Visual Analog Scale
WOMAC: Western Ontario and McMaster University Osteoarthritis Index
